# Socioeconomic inequalities in life expectancy and disability-free life expectancy among Chilean older adults: evidence from a longitudinal study

**DOI:** 10.1186/s12877-021-02126-9

**Published:** 2021-03-11

**Authors:** Ximena Moreno, Lydia Lera, Francisco Moreno, Cecilia Albala

**Affiliations:** 1 Instituto de Nutrición y Tecnología de los Alimentos, Universidad de Chile, Avenida El Líbano 5524, Macul, Santiago, Chile; 2 Keiser University, 1900 W Commercial Blvd, Fort Lauderdale, FL 33309 USA; 3 Universidad de Santiago de Chile, Avenida Libertador Bernardo O’Higgins, 1611 Santiago, Chile

**Keywords:** Life expectancy, Disability-free life expectancy, Ageing, Socioeconomic inequalities

## Abstract

**Background:**

Chile has one of the longest life expectancies of Latin America. The country is characterised by an important macroeconomic growth and persisting socioeconomic inequalities. This study analyses socioeconomic differences in life expectancy (LE) and disability-free life expectancy (DFLE) among Chilean older people.

**Methods:**

The sample of the Social Protection Survey, a longitudinal study, was analysed. Five waves, from 2004 to 2016, were considered. The indicator was disability, defined as having difficulties to perform at least one basic activity of daily living. Type of health insurance was used to determine socioeconomic position (SEP). Total LE and DFLE were estimated with multistate life table models.

**Results:**

At age 60, men in the higher SEP could expect to live 3.7 years longer (22.2; 95% CI 19.6–24.8) compared to men of the same age in the medium SEP (18.4; 95% CI 17.4–19.4), and 4.9 years longer than men of the same age in the lower SEP (17.3; 95% CI 16.4–18.2). They also had a DFLE (19.4; 95% CI 17.1–21.7) 4 (15.4; 95% CI 14.6–16.1) and 5.2 (14.2; 95% CI 13.4–14.9) years longer, compared to the same groups. Women aged 60 years in the higher SEP had a LE (27.2; 95% CI 23.7–30.8) 4.6 (22.7; 95% CI 21.9–23.5) and 5.6 (21.6; 20.6–22.6) years longer, compared to women in the medium and the lower SEP. The difference in DFLE, for the same age and groups was 4.9 and 6.1 years, respectively (high: 21.4; 95% CI 19.5–23.3; medium: 16.5; 95% CI 15.8–17.1; low: 15.3; 95% CI 14.6–16.0). Socioeconomic differences in LE and DFLE were observed among both sexes until advanced age.

**Discussion:**

Socioeconomic inequalities in LE and DFLE were found among Chilean older men and women. Older people in the highest SEP live longer and healthier lives.

**Conclusion:**

A reform to the Chilean health system should be considered, in order to guarantee timely access to care and benefits for older people who are not in the wealthiest group.

## Background

Life expectancy (LE) in Latin America has been growing at a faster pace, compared to North America and Europe [1]. Chile is one of the countries with the highest LE at birth (80.3) of the región [2]. Ageing of the Chilean population has also been fast, with an ageing index of 87.2, considering an average of 51.8 for the región [2]. Apart from LE, it is important to know what proportion of those years will be lived in good health. Disability is among the most widely used indicators to assess health status of the older population, and to estimate disability-free life expectancy (DFLE) [3]. It has been reported that Chilean older women could expect to live more years disabled, compared to men of the same age [4, 5].

There is evidence that along with the fast economic growth experienced by the country during the last decades, public policies addressing socioeconomic inequalities have been insufficient [6]. Furthermore, the organization of the health system, characterised by the coexistence of a public health insurance programme and market-based private health insurance programmes, has deepened unequal access to health care [7]. Whereas in the public health system the employee contribution is based on income, in the private insurance system the contribution depends on individual health risk and number of dependents [7]. Private health insurance companies are allowed to create barriers to the affiliation of poorer and older people, by increasing premiums, deductibles and copayment, considering pre-existing conditions and health status [8]. Therefore, older people who are affiliated to the private health insurance system, are a selected group with a high income for the standard of the country, able to afford all these costs [8, 9]. In 2017, 84.9% of the Chilean population aged 60 or more years was affiliated to the public health insurance system [10]. Previous studies have used the type of health insurance as a socioeconomic indicator, finding important differences in health and functional status among Chilean older people, depending on their socioeconomic position [11, 12]. Recent analyses have reported marked socioeconomic inequalities in LE at old age in Chile [13, 14]. To date, LE in a specific health status by socioeconomic position among Chilean older people has not been analysed. The aim of this study was to analyse socioeconomic differences in life expectancy and disability-free life expectancy among Chilean older people.

## Methods

### Sample

This study was based on secondary analyses of data from the Social Protection Survey (SPS). The SPS is aimed to collect information about social security in Chile, which includes a brief section about health status [15]. It is designed as a fixed panel plus births longitudinal study [15]. The baseline sample in 2002 was representative of people affiliated to the Chilean pension system. In 2004, people not affiliated to the pension system and a refreshment sample were included, and the sample became nationally representative of the Chilean population aged 18 years or more [16]. The sample was recruited using a multi-stage stratified cluster sampling, considering the smallest territorial divisions as the clusters. The sampling method used to expand the sample in 2004 was similar. Since the sample from 2004 was nationally representative and disability started to be measured that year, this wave was the baseline measurement in our analyses, including people aged 60 or more years. A total of 3286 people had data on disability, type of health insurance and at least one follow-up measurement. Four succesive waves were considered (2006, 2009, 2012, 2016). The data collection of the last wave of the SPS finished in July 2016 [17].

In the context of the present study, no ethical approval was required, since secondary analyses of anonymised and publicly available databases were performed.

### Outcome measures

To estimate DFLE, disability was ascertained at each wave by asking participants if they usually needed help or had difficulties to perform basic activities of daily living, including bathing, dressing, eating and getting out of bed. A person that reported difficulties with at least one of these activities was classified as having a disability.

Mortality was ascertained via retrospective measurement during the household interview. The information on dates of death collected in the SPS was validated using official administrative data on the pension system history of active members, pensioned and deceased to July 2016.

### Socioeconomic indicator

Health insurance type was used as socioeconomic indicator. People affiliated to the private health insurance system were considered as in high socioeconomic position (SEP). The medium socioeconomic position included people affiliated to the public health system who had received or still received a salary during their working life or contributed to the system to be affiliated (groups B, C and D). People who were affiliated to the system in group A (without income, hence not able to contribute), were classified as in low socioeconomic position.

### Statistical analyses

Baseline characteristics of the sample by sex and SEP were reported. Chi square test and one-way ANOVA were used to determine SEP differences.

The observation period was 15.7 years (from November 2004 to July 2016). All participants that had at least one follow-up measure were included in the analysis (*n* = 3286). A total of 13,280 records were available.

A three state model was defined. State 1 was ‘healthy’ (without disability), state 2 was ‘unhealthy’ (with disability), and state 3 was ‘dead’. Since ‘dead’ was an absorbing state, there were four possible transitions between these states, namely: healthy to unhealthy, unhealthy to healthy, healthy to dead and unhealthy to dead. When the state between two known states was missing, interval censoring was used. Right censoring was used when the health state at the end of follow-up was unknown, but alive. MSM for R [18] was used to fit multi-state Markov models, in order to estimate the different expected health transitions, under the assumption of future evolution depending on the current state. A transition intensity matrix, Q, was calculated with all the observations, reflecting the instantaneous risk of moving from one state to another. Age (time-varying), sex and SEP were defined as covariates. To estimate total and marginal LEs, the ELECT (Estimating Life Expectancies in Continuous Time) package for R [19] was used. In order to do so, ELECT fits multinomial logistic regression models for state prevalence.

Total, healthy and unhealthy LEs for men and women at age 60, 70 and 80, in each SEP, were estimated separately. To determine differences in these estimates, 95% confidence intervals were calculated. R version 4.0.3 was used for statistical analyses.

## Results

Table 1 presents baseline characteristics for men and women in different SEP. A greater proportion of men and women in the higher SEP were in the youngest age group, and they were less likely to be in the oldest age group. Disability prevalence increased as SEP decreased. The number of respondents in each wave of the study was 3183 in 2006, 2844 in 2009, 1618 in 2012, and 2196 in 2016.

**Table 1:** Baseline characteristics of the sample by sex and socioeconomic position

	High (*n* = 251)^a^	Medium (*n* = 2074)^a^	Low (*n* = 961)^a^	*P* value
Men (*n* = 1640)				
Age (mean, SD)	66.2 (5.7)	70.2 (7.5)	69.6 (7.9)	< 0.001
Age group (%)				
60–69	74.1	51.9	56.2	< 0.001
70–79	23.1	35.7	31.7	
80 or more	2.8	12.5	12.1	
Disability (%)				
Yes	2.8	11.5	15.2	< 0.001
No	97.2	88.5	84.8	
Women (*n* = 1646)				
Age group (%)				
Age (mean, SD)	68.5 (7.7)	70.7 (7.9)	71.5 (8.8)	0.02
60–69	63.0	49.9	50.3	0.002
70–79	25.0	34.8	30.8	
80–89	12.0	15.3	18.9	
Disability (%)				
Yes	7.4	19,2	24.9	< 0.001
No	92.6	80.8	75.2	

^a^ Column percent totals may be greater than 100% due to rounding

By the end of the follow-up, 34.1% (1119) participants had died. With respect to the number of deaths within groups, 41 (16.3%) participants in the higher SEP, 715 (34.5%) in the medium SEP, and 363 (37.8%) in the lower SEP, died during the observation period. The number of incident disability cases was 1371, and recovery from disability occurred 689 times. As observed in Table 2, in the case of men at age 60, those in the highest SEP expected to live 3.8 and 4.9 more years, compared to men in the medium and the lower SEP, respectively. These differences were present until 80 years of age, with a 2.3 and 2.9 years longer LE among me in the lower SEP, compared to men in the other SEP. At 60 years of age, women in the highest SEP expected to live 4.6 more years than women in the medium SEP, and 5.6 more years, compared to women in the lower SEP.

**Table 2:** Total life expectancy, disability-free life expectancy and disabled life expectancy for men and women, by socioeconomic position

	High	Medium	Low
Men			
60 years			
TLE	22.2 (19.6–24.8)	18.4 (17.4–19.4)	17.3 (16.4–18.2)
DFLE	19.4 (17.1–21.7)	15.4 (14.6–16.1)	14.2 (13.4–14.9)
DLE	2.8 (1.5–4.0)	3.1 (2.7–3.4)	3.1 (2.7–3.6)
70 years			
TLE	14.8 (12.4–17.2)	11.7 (11.2–12.2)	10.9 (10.0–11.7)
DFLE	12.4 (10.5–14.3)	9.2 (8.8–9.6)	8.4–7.8-8.9)
DLE	2.4 (1.3–3.4)	2.5 (2.2–2.8)	2.51 (2.1–3.0)
80 years			
TLE	9.1 (7.2–10.9)	6.8 (6.3–7.3)	6.2 (5.7–6.7)
DFLE	7.3 (6.0–8.6)	5.1 (4.7–5.4)	4.5 (4.1–4.9)
DLE	1.8 (0.8–2.8)	1.7 (1.4–2.0)	1.7 (1.3–2.1)
Women			
60 years			
TLE	27.2 (23.7–30.8)	22.7 (21.9–23.5)	21.6 (20.6–22.6)
DFLE	21.4 (19.5–23.3)	16.5 (15.8–17.1)	15.3 (14.6–16.0)
DLE	5.8 (3.2–8.5)	6.2 (5.7–6.8)	6.4 (5.5–7.3)
70 years			
TLE	19.2 (16.3–22.0)	15.2 (14.4–16.0)	14.4 (13.3–15.4)
DFLE	13.9 (11.9–15.9)	9.9 (9.5–10.3)	9.0 (8.4–9.6)
DLE	5.2 (2.9–7.6)	5.3 (4.7–5.8)	5.3 (4.4–6.2)
80 years			
TLE	12.5 (10.2–14.8)	9.4 (8.7–10.0)	8.9 (8.1–9.5)
DFLE	8.3 (6.8–9.8)	5.5 (5.1–5.9)	4.9 (4.6–5.3)
DLE	4.2 (1.9–6.4)	3.9 (3.3–4.5)	3.9 (3.3–4.4)

*TLE* total life expectancy; *DFLE* disability-free life expectancy; *DLE* disabled life expectancy

Men aged 60 in the highest SEP expected to live 4 and 5.2 more years free of disability, compared to men of the same age in the medium and the lowest SEP. With respect to DFLE at age 60, women in the highest SEP had an advantage of 4.9 and 6.1 years, compared to women in the medium and lower SEP, respectively.

**Fig. 1:**
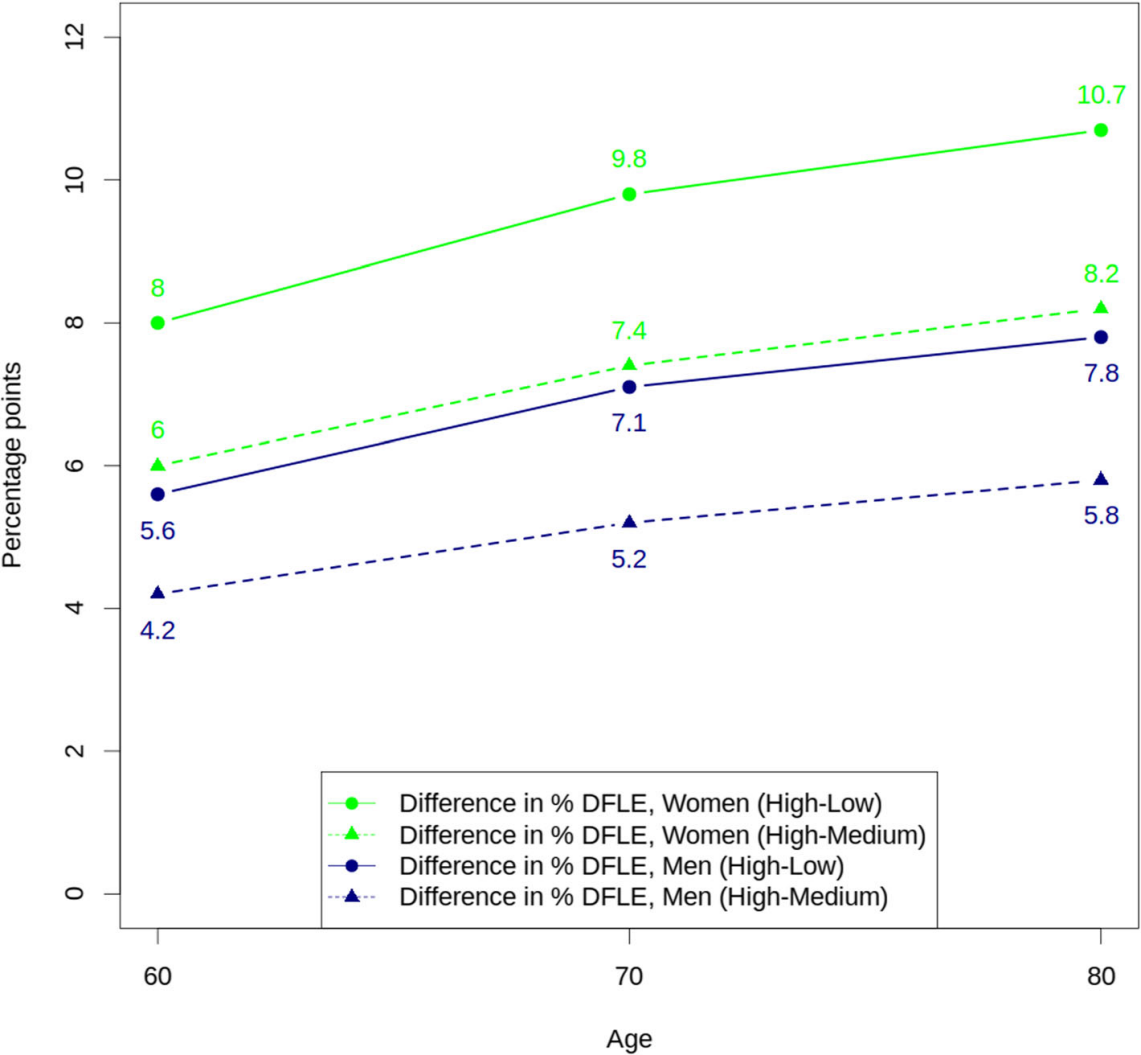
Difference in the proportion of years to be lived free of disability, by sex

Differences in DFLE between people in the highest SEP and people in the medium and the lower SEP were observed until 80 years of age. At this age, these differences reached 2.2 and 2.8 more years free of disability for men in the highest SEP, compared to the other SEP, and 2,8 and 3,4 more years for women in the highest SEP, compared to women in the other SEP. Although the absolute difference in years free of disability decreased with age, the difference in the proportion of years to be lived free of disability increased at older ages (Fig. 1).

Although women in the medium and the lowest SEP had a longer total LE compared to men of the same SEP, their LE free of disability was not longer. Differences in LE and DFLE were observed among men and women.

## Discussion

Our results confirm that the trajectories of health, disability and mortality are more adverse for Chilean older people who are not in the highest SEP. We found significant differences in LE, depending on SEP, among Chilean older men and women. Comparing the highest and the lowest SEP, at 60 years the difference was 4.9 years for men, and 5.6 years for women, in favour of the wealthiest group. These differences are marked, but more conservative than those found in previous studies about the Chilean population [13, 14]. This could be due to the fact that the data analysed in previous studies allowed them to make finer comparisons, such as the difference between the first and the tenth decile [14]. In that case, the difference between the poorest and the richest decile reached 7.7 years for men aged 60, and 17.8 years for women of the same age.

People in the better-off group also expected to live more years free of disability, compared to people in the medium and lowest SEP. Since our indicator of disability was having difficulties or needing assistance to carry out basic activities of daily living, our results indicate that Chilean older people in the medium and the lowest SEP can expect to live more years of dependency. Less years free of disability have an impact on health, well-being and finances at a family level [20, 21] and are associated with greater demand for social and health care [22].

As observed in a previous study comparing population from England and the United States [23], the absolute difference between LE and DFLE by SEP decreased at older ages. However, in our study the difference in the proportion of years to be lived with disabilities, between the highest and the lower SEP, increased with age.

In Chile, the type of health insurance is an expression of socioeconomic segregation among Chilean older people, particularly between the richest group and the rest of the population. Although the socioeconomic inequalities in LE and DFLE observed are associated with multiple factors through the life-course, the organisation of the Chilean health system and health insurance scheme themselves are a source of health inequalities, as previous studies have discussed [7, 8]. Those who have access to private health insurance are not only richer, but also healthier, whereas the older population affiliated to the public health insurance are more likely to have more health problems and risks [24]. In 2004, 7.5% of people aged 60 or more years were affiliated to the private health insurance [25]. Around that time, 70% of the people affiliated to the private health insurance were under 50 years [26]. Since the private health insurance premiums increase progressively with age and health risks [24], the proportion of older people affiliated to the private health insurance continues to decrease, as observed in our study and reported previously [27]. According to this, a proportion of people in our sample who were affiliated to the private health insurance system at baseline, probably migrated to the public health insurance, due to a limited payment capacity, health problems or both. If so, we would be underestimating the differences between the wealthiest group of older people in Chile – those who are able to stay in the private health insurance system until a very old age – and the rest of the population.

Even though the Chilean public health system guarantees free access to health care for an important number of conditions, people affiliated to the private health insurance have more access to specialised medical services, laboratory tests and surgery [7]. In practice, the public health system is underfunded and insufficiently equipped, resulting in long waiting lists for specialist treatment and surgery, affecting more acutely the older, the poor and those with chronic conditions [28]. As previous reports have stressed [6, 7, 29], it is urgent to develop public policies able to meet the social and health needs of an increasingly older population in Chile.

Previous studies have analysed two models that could explain social inequalities in health. One of them is social causation, which considers that socioeconomic position determines health during the life-course, whereas health selection considers that social mobility depends on the health status [30]. We did not have historical data that allow us to analyse health trajectories and social mobility during the life-course, hence we could not measure and cannot rule out the effect of health status on socioeconomic position. However, there are several elements that support the hypothesis that socioeconomic position is a determinant of health inequalities in life expectancy and disability-free life expectancy among older people in Chile. First, European research suggests that the social causation has a higher importance to explain health inequalities in old age [30, 31]. Second, Chile is one of the most unequal countries in the world [32], with a pattern of high concentration in a reduced group at the top and strong vertical barriers to social mobility [33].

A strength of this study is that it analysed longitudinal data and used multistate models, in order to take into account health transitions and time spent in each health state. For the first time, socioeconomic inequalities in DFLE among Chilean older people have been explored.

There are some limitations to be considered. Although the sample of the SPS was nationally representative of Chilean people aged 18 years or more, people with missing data and those who have only one observation could not be included in the analyses. This could have affected representativeness and should be taken into account when interpreting the results. In fact, according to national data, in 2004 LE for men aged 60 was 19.7 years, and for women of the same age, 23.9 years. LE estimated with our data were 18.8 (95% CI 18.3–19.3) years for men, and 23.0 (95% CI 22.2–23.8) years for women. Also, the sample size affected the precision of our estimates. Therefore, the observed differences between socioeconomic positions could be smaller or larger than expressed by point estimates. Since a small proportion of older people are affiliated to the private health insurance, the sample size for this group was the smallest, resulting in less precise estimates. Nevertheless, the differences found in LE and DFLE between this SEP and the other groups were significant. As mentioned above, we were not able to take into account migrations from the private to the public health insurance system as age increased, which might have led to an underestimation of differences in LE and DFLE between the higher and the other SEP. Also, the indicator used to determine SEP does not allow to make direct comparisons with international studies. However, type of health insurance is a good indicator of SEP among Chilean older people, since it is associated with education and income [11, 34].

## Conclusion

Socioeconomic inequalities in LE and DFLE were found among Chilean older men and women. Older people in the highest SEP live longer and healthier lives. Our results support previous analyses that highlight the insufficient capacity of public policies in Chile to meet the social and health needs of an increasingly older population. A reform to the Chilean health system should be considered, in order to guarantee timely access to care and benefits for older people who are not in the wealthiest group.

## Data Availability

The datasets generated and/or analysed during the current study are available in the database repository of the Social Protection Undersecretary from the Ministry of Labor and Social Security: https://www.previsionsocial.gob.cl/sps/biblioteca/encuesta-de-proteccion-social/bases-de-datos-eps/
